# Seromolecular and histopathological study on *Toxoplasma gondii* infection in ruminants in Aswan, Egypt

**DOI:** 10.1186/s12917-025-05195-9

**Published:** 2025-12-30

**Authors:** Ahmed Gareh, Nady Kh. Elbarbary, Ahmed Fotouh, Ahmed Maher, Yasser M. Mohamed, Mohammed F. Ragab

**Affiliations:** 1 Parasitology Department, Faculty of Veterinary Medicine, Aswan University, Aswan, 81528 Egypt; 2 Food Hygiene and Control Department, Faculty of Veterinary Medicine, Aswan University, Aswan, 81528 Egypt; 3 Pathology and Clinical Pathology Department, Faculty of Veterinary Medicine, New Valley University, El-Kharga, Egypt; 4 Animal Medicine Department (Infectious Diseases), Faculty of Veterinary Medicine, New Valley University, El-Kharga, 72511, Egypt; 5 Medical Parasitology Department, Faculty of Medicine, Assiut University, Assiut, Egypt; 6 Medical Parasitology Department, Faculty of Medicine, Damietta University, Damietta, 34517 Egypt

**Keywords:** *Toxoplasma gondii*, NPCR, Cattle, Buffalo, Sheep, Goats, Seroprevalence

## Abstract

**Background:**

*Toxoplasma gondii* is a globally distributed zoonotic parasite that affects both humans and animals, with significant implications for public health and livestock production. The current research aims to update the information on the present prevalence of *T. gondii* and the risk factors associated with the infection in domestic ruminants in Aswan, Egypt, from August 2024 to January 2025, using serological, histopathological, and molecular approaches.

**Methods:**

The blood of 387 domestic ruminants collected during the antemortem examination from four central abattoirs in the Aswan governorate, Upper Egypt, was inspected for the occurrence of anti-*T. gondii* antibodies through a modified agglutination technique. Data were confirmed by a nested polymerase chain reaction that targeted *T. gondii* DNA (*B1* gene). Tissue specimens (heart and diaphragm) from seropositive animals were collected during postmortem examination and subjected to a histopathological and immunohistochemical approach.

**Results:**

The overall occurrence of *T. gondii* was 29.5% (114/387), with seropositivity of 33.5% (52/155), 28.2% (22/78), 23.6% (21/89), and 29.2% (19/65) in cattle, buffalo, sheep, and goats, respectively. The studied risk factors (age, gender, breed, body condition, and location) in this study were detected to be significantly related to the presence of *T. gondii* infection (*p* ˂ 0.05). Histopathological examination detected tissue cysts in 38 out of 114 cardiac muscles of seropositive animals and failed to detect any cysts in the diaphragm tissue, indicated by encased, circular to elongated, basophilic cysts with many bradyzoites entrenched in muscle fibers by H&E staining, while showing intense brown granule staining of lymphoblastic cells by immunohistochemistry assay. Nested PCR confirmed the presence of the *B1* gene of *T. gondii* in blood samples of all seropositive animals (100%).

**Conclusions:**

The combined use of serology, PCR, and IHC demonstrates that *T. gondii* is present in slaughtered ruminants in Aswan and that viable tissue cysts are present in edible tissues. These findings highlight a potential risk of zoonotic transmission through the consumption of undercooked meat and emphasize the need for monitoring and control measures to reduce the burden of foodborne toxoplasmosis in Egypt.

**Supplementary Information:**

The online version contains supplementary material available at 10.1186/s12917-025-05195-9.

## Introduction

*Toxoplasma gondii* (*T. gondii*) is a foodborne zoonotic coccidian protozoan that has a worldwide distribution, infecting humans, ruminants, and other warm-blooded animal species [1]. The final hosts belong to the Felidae family, where the sexual stage takes place. This protozoan develops an asexual cycle in the tissues of various warm-blooded species, including humans, which act as intermediate hosts [2]. The environmental transmission of *T. gondii* is maintained mainly through oocysts shed by definitive feline hosts, while intermediate hosts contribute to the parasite’s life cycle by harbouring tissue cysts that can infect new hosts when consumed. There are three main ways that definitive and intermediate hosts can become infected: by swallowing sporulated oocysts from contaminated food and water, by eating undercooked meat that has bradyzoite tissue cysts, or by vertical transmission that occurs congenitally, when tachyzoites cross the placenta from infected mothers to their offspring [3].

Ruminant animals are especially sensitive to *T. gondii* infection, which is regarded as one of the leading causes of abortion, stillbirth, and weak offspring in sheep and cattle [4]. Many researchers worldwide have investigated the prevalence of *T. gondii* in various animal meats, as well as the significance of certain species as a cause of infection in humans, varying by location and culture [1, 5, 6]. However, *T. gondii* was found in 25% of in ruminant meat from Malaysia [7]; it was 47% overall in ruminant blood from Turkey [8]; it was 11.6% overall in liver and diaphragm tissues of slaughtered sheep and goats from Iran [9]; the rate was 13.0% in diaphragm and heart tissues of slaughtered cattle from Poland [10]; it was 63.3% in serum and milk of goat from Italy [11]; and it was found in 8.3% and 13.3% in sera of beef cattle and goats from China [12]. Additionally, Table 1 indicates that *T. gondii* has been detected in multiple Egyptian provinces. Ruminant animals are more likely to become infected because they graze in areas contaminated with oocysts. Therefore, the high occurrence of *T. gondii* detected in ruminants globally impacts livestock production economics and poses a considerable zoonotic health risk to humans who consume undercooked or raw meat from infected animals [7].

Toxoplasmosis has been demonstrated to induce fever, lymphadenopathy, headache, myalgia, arthralgia, vertigo, and, in severe cases, encephalitis, blindness, and abortions in humans [7]. Laboratory diagnosis of *T. gondii* relies on a combination of biological, histological, serological, and molecular techniques [13]. While serological techniques are essential for assessing exposure to T. *gondii*, they do not always reflect current infection status. The incorporation of molecular detection methods, such as PCR, enables the direct identification of parasite DNA in various biological samples and complements serological results [14]. The combined application of these methods provides a more accurate and holistic picture of the epidemiology of *T. gondii*, which is critical for both public health and veterinary contexts.

In Egypt, traditional husbandry systems are often based on smallholders who raise multiple animal species, including cattle, buffalo, sheep, and goats, sometimes in proximity to each other [4]. Previous reports also noted the frequent presence of free-roaming cats around rural farms, which can contribute to environmental contamination with *T. gondii* oocysts [15]. Sanitary and hygienic conditions may be suboptimal in many of these settings, particularly in low-income communities [16]. Regular updates on the prevalence of infectious illnesses are crucial for implementing successful infection-control strategies. Therefore, it is essential to assess ruminant exposure to *T. gondii* as well as in meats intended for human consumption in Egypt. Consequently, the objective of this investigation was to determine the prevalence of *T. gondii* among slaughtered ruminants in Aswan Governorate, to assess risk factors associated with seropositivity, and to confirm infection using serological, molecular, and histopathological methods.

**Table 1:** Seroprevalence of *T. gondii* in ruminants in Egypt (2015–2025)

Governorate	Year	Host	Samples	Technique	Frequency %	Reference
Dakahlia	2015	Sheep	Blood, Tissues, Fecal matter	LAT/IHA/ELISA	41.7/66.1/62	[17]
		Goat			49.4/64.2/50.6	
Assuit	2015	Sheep	Milk	LAT	39.7	[18]
		Goat			38.3	
Qena/Sohag	2016	Cattle	Blood	LAT/ELISA	29.2/28.2	[19]
Quena, Kafr El Sheikh & Minoufiya		Sheep			47.8/51.4	
		Goat			35.1/39.4	
Assuit	2016	Cattle	Blood	LAT/ELISA	32.1/73.2	[20]
		Buffalo			74.5/20	
		Sheep			44.0/86	
		Goat			47.4/87.7	
Cairo, Giza & Kalubiya	2017	Buffalo	Blood, Tissues	ELISA	17.1	[21]
		Cattle			35.5	
		Sheep			64.2	
		Goat			43.3	
Cairo, Giza & Al-Sharkia	2018	Sheep	Blood, Tissues	ELISA/OnSite Toxo IgG/IgM Rapid test cassettes	51.3/58.4	[22]
		Goat			41.0/45.2	
Cairo, Giza, Dakahlia & Sharkia	2018	Sheep	Blood	IFA/ELISA	4.1/26	[23]
		Goat			62.0	
Menoufia	2021	Buffalo	Blood	ELISA	8.2	[24]
Alexandria & Matrouh	2022	Sheep	Blood	ELISA	43.8	[25]
		Goat			27.9	
		Cattle			13.5	
Dakahlia	2022	Sheep	Milk	ELISA	66.7	[26]
Dakahlia, Beni Suef, Qena & Red Sea		Sheep			35.6	
Dakahlia		Buffalo			0.0	
Qena, Cairo, Sohag, Dakahlia		Cattle			2.4	
Luxor	2022	Sheep	Blood	ELISA	40.2	[27]
		Goat			34.8	
Beheira	2023	Cattle	Blood	ELISA	5.3	[28]
Sohag	2023	Buffalo	Blood	MAT	58.6	[4]
		Cattle			59.4	
		Goat			46.0	
		Sheep			38.8	

*LAT* Latex agglutination test, *IHA* Indirect hemagglutination, *ELISA* Enzyme-linked immunosorbent assay, *IFA* Indirect fluorescent antibody test, *MAT* Modified agglutination test

## Materials and methods

### Research plan

**Fig. 1:**
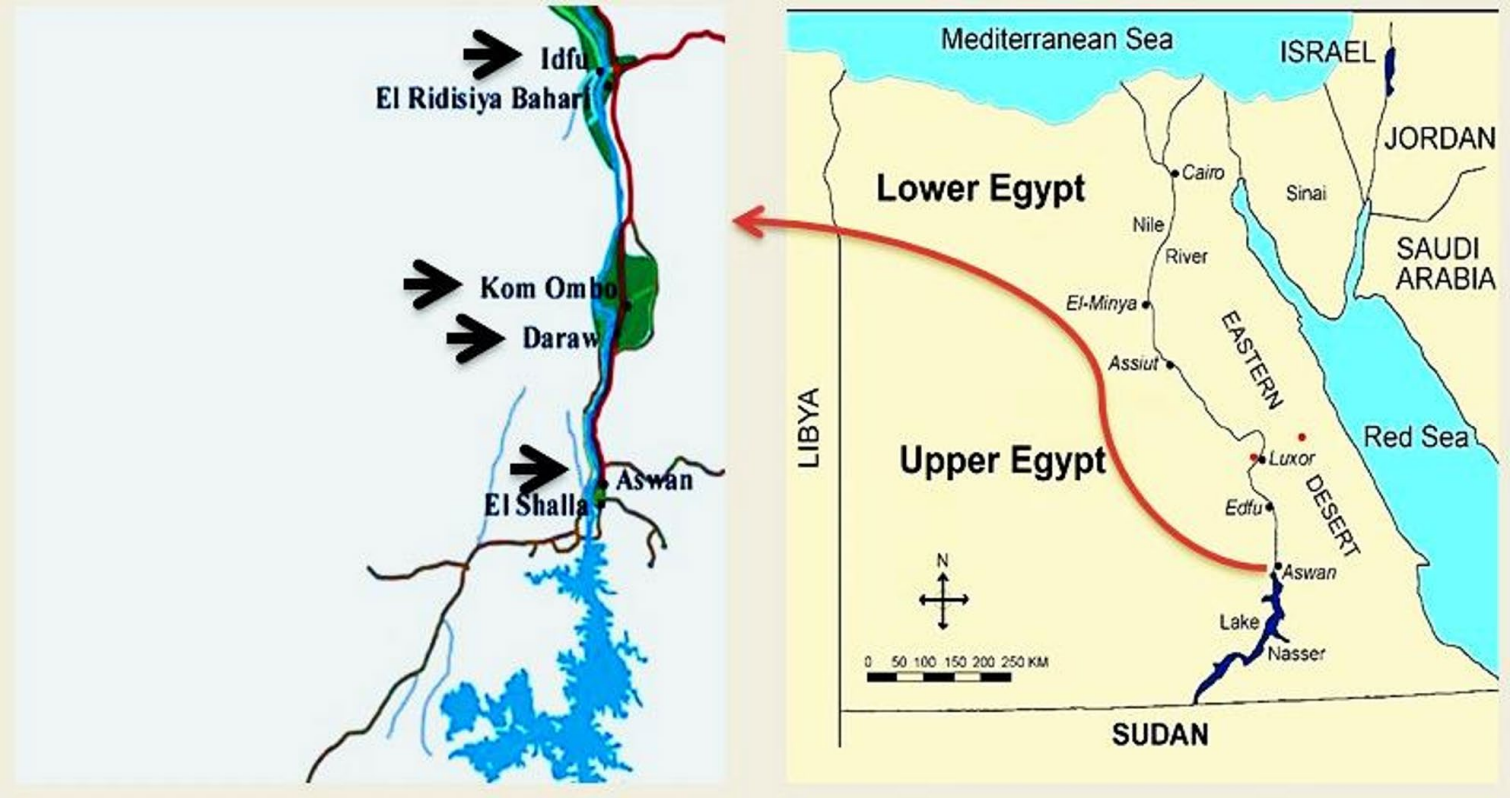
Sampling sites (arrow head), Aswan map, Egypt

A cross-sectional investigation was conducted from August 2024 to January 2025, involving the selection of central abattoirs in Aswan, Egypt (Fig. 1). The governorate of Aswan spans a total territory of 62,726 km², of which 12,203 km² are populated. It is situated on the east bank of the Nile, close to the first cataract, slightly north of the Aswan Dam, at latitudes 24° 5′ 20.1768″ N and 32° 53′ 59.3880″ E in southern Egypt. Aswan’s summers can reach temperatures of over 41 °C and are extremely dry, while the winters are pleasant, with average temperatures of 26 °C and little precipitation. The sampling sites were chosen due to the abundance of fresh meat from locally slaughtered animals in the research area, as well as the high yearly cattle throughput. All of the animals that were slaughtered had thorough pre- and postmortem clinical examinations. Information regarding slaughtered animals, including breed, age, gender, and place of origin (local or imported), was obtained from the abattoir database records.

### Sample size

The sample size was determined following Thrusfield [29] with a 95% CI and a 5% absolute precision. Thus, Hussein et al. [5] calculated the anticipated prevalence of *T. gondii* to be 18%. According to the following formula: $\:n=\frac{{Z}^{2}\times\:{P}_{exp}\left(1-{P}_{exp}\right)}{{d}^{2}}$

n = required sample, Z = appropriate percentage for the standard deviation for the expected confidence = 1.96, P_exp_ = predictable occurrence, and d = anticipated total precision (usually 0.05).

$\:n=\frac{{1.96\:}^{2}\times\:0.18\left(1-0.18\right)}{{0.05}^{2}}=226.8\:$(Minimum sample size)

as a result, 387 samples from various animal species were examined for the presence of *T. gondii*, with the larger sample size increasing the likelihood of detecting positive cases.

### Animals

About 387 samples were gathered from different domestic ruminant species (155 cattle, 78 buffaloes, 89 sheep, and 65 goats) intended for slaughter in different central abattoirs in the Aswan governorate, as stated by Egyptian veterinary legal regulations (law No. 2128/2011) [30]. The breeds of animals for the cattle were native breeds (Baladi and Hagien) and imported breeds (Sanga, Friesian, and Holstein) from Sudan and Ethiopia in Africa; for the buffaloes, they were Egyptian (Riverine) Buffalo; for the sheep, they were Saidi and Ossimi; and for the goats, they were Baladi, Saidi, and Zaraibi or Nubian. Cattle and buffaloes were aged between 2 and 10 years, while sheep and goats were aged between 1 and 5 years. In accordance with the dental eruption stages, the animals were classified as young (< 2 years), mature (2–5 years), and elderly (> 5 years). Animals were classified as poor, medium, or good according to their body condition score. Poor animals exhibited hidebound bones and deep-sunk tail bases, while medium animals had visible ribs and other prominent bony features but a fair, fleshy background when palpated. Good animals had skeletal structures that were only palpable [31].

### Blood sampling

Blood samples were obtained throughout the antemortem examination of the investigated animals in the abattoir. From each animal, 15 mL of blood (10 mL of blood in plain tubes without anticoagulant was used for serology assessment, and 5 mL of blood with EDTA as anticoagulant was used for PCR confirmation) was acquired by piercing the jugular vein with sterile syringe needles. Every sample was stored in portable coolers with polythene ice packs and delivered to Aswan University’s Department of Food Hygiene. Sera were extracted by centrifugation at 1,107 rcf for 15 min. The supernatant was moved to a fresh Eppendorf tube and stored at −20 °C until testing [32].

### Tissue sampling

Tissue (heart and diaphragm) specimens, which represented the most predilection sites for *T. gondii*, were obtained from the carcass during the routine postmortem inspection in the abattoir under the supervision of the official veterinarian inspector. The ground meats were excluded to avoid the combination of meat from different animal origins. Tissue samples were kept at −20 °C until histopathological analysis was completed (2–5 days).

### Serological assessment

The serum specimens were serologically tested for the detection of anti-*T. gondii* antibodies, particularly IgG, employing the modified agglutination test (MAT, cut-off titer ≥ 1:25), which involves formalin-fixed tachyzoites as previously published [33]. The antigen used for the modified agglutination test (MAT) was supplied by the Zoonotic Disease Laboratory at the National Research Centre, Cairo, Egypt, in collaboration with the Toxoplasmosis Research Unit in Reims, France. Sera with titers > 1:25 were declared positive, meeting the accepted cut-off for *T. gondii* seropositivity [4]. Sera that tested positive at a dilution of ≥ 1:25 were retested at 1:25, 1:50, 1:100, and ≥ 1:500 concentrations for endpoint titer, which represents the highest dilution at which antibodies against *T. gondii* could still be detected.

### Histopathological findings

Fixing was performed on tissue samples obtained from seropositive animals using neutral buffered formalin 10% dehydrated in increasing alcohol concentrations (70%, 80%, 90%, and 100%). Samples were immersed in paraffin wax, and 4–5 μm-thick transverse sections were cut using a microtome and placed on glass slides. The samples were normally treated in paraffin for light microscopy, and histological slices were prepared for hematoxylin-eosin (H&E) staining [34]. Each sample was extensively reviewed by skilled pathologists from New Valley University’s Faculty of Veterinary Medicine. The cysts were reported qualitatively, and the findings were collected and documented with a Canon digital camera (Canon Powershot A95) connected to a Leitz Dialux 20 Microscope (Germany).

### Immunohistochemical staining (IHC)

The occurrence of *T. gondii* tissue cysts has been confirmed in IHC-stained diaphragm and heart seropositive individuals. The slices were deparaffinized and hydrated, and endogenous peroxidase was inhibited using a 3% hydrogen peroxide solution. Antigen recovery was achieved by incubating the sections in a 96 °C water bath for 30 min. The slices were incubated for 30 min in milk and 10% bovine serum albumin solution to prevent nonspecific binding. The sections were then treated for 30 min with a 1:200 diluted primary rabbit anti-*T. gondii* antibody (Neomarkers, Fremont, CA, USA). As indicated by the manufacturer, the pieces were treated with DAKO LSAB (DAKO Corp., Carpinteria, CA, USA). Diaminobenzidine (DAB; DAKO Corporation, Carpinteria, CA, USA) was employed as the chromogen to illustrate the parasite’s life cycle stages. Harris hematoxylin was employed to counterstain all samples. Bradyzoites stained brown with DAB (3,3′-diaminobenzidine) were regarded as positive [35].

### Molecular assay

#### DNA extraction

About a 0.05–1 mL venous blood sample of each seropositive ruminant with EDTA was used for DNA extraction using the Quick-gDNA™ MiniPrep kit (Cat. No. D3024, Zymoresearch, USA), as mentioned by the manufacturer’s instructions for DNA purification from blood. The DNA samples were maintained at a temperature of −20 °C until investigation [36].

#### Nested polymerase chain reaction (nPCR)

The *T. gondii B1* gene was detected using conventional nested nPCR with a specific primer sequences (W1020300X, Willowfort Co., UK) [9], which is also listed in Table 2. A NanoDrop spectrophotometer (Thermo Scientific, ND8000) was employed to determine the concentration of DNA, which ranged from 25 to 100 ng/µL. For the first round of PCR, the following components were assembled: 0.5 µM primers, 200 µM dNTPs, 1.5 mM MgCl₂, 1.5 units of Amplitaq polymerase, and 10 µL of DNA template, with a total volume of 50 µL. Similar to the first round, the second-round amplification reaction employed 10 µL of the previous round’s PCR product as a template, with a total volume of 50 µL. Positive controls consisted of *T. gondii* isolates obtained from cattle that had been previously confirmed by PCR and genotyped as Type II, and were maintained at the Animal Health Research Institute in Cairo, Egypt. The ddH2O was used for the negative control. The PCR yield was loaded onto a 3% agarose gel with SYBR Safe DNA gel dye (Invitrogen, USA), run at 100 V for 30 min, and visualized using a transilluminator (GEL DOC XR).

**Table 2:** Primer sequences and PCR conditions for detection of *T. gondii B1* gene

Primer	Sequence (5′- 3′)	Size	PCR conditions
First round (Outer primer)	F: GGAACTGCATCCGTTCATGAG	200 bp	95 °C for 5 min followed by 40 cycles (95 °C/30 s, 56 °C/30 s, 72 °C/30 secs)
	R: TCTTTAAAGCGTTCGTGGTC		
Second round (Inner primer)	F: TGCATAGGTTGCAGTCACTG	100 bp	95 °C for 5 min followed by 35 cycles (95 °C/30 s, 56 °C/30 s, 72 °C/30 s)
	R: GCGACCAATCTGCGAATACACC		

### Statistical analysis

SPSS version 16.0 was used for statistical analysis, and the associations between potential risk factors (age, gender, breed, body condition, and location) and serostatus were assessed using the chi-square test (χ^2^). Serostatus was defined by the presence or absence of anti-*T. gondii* IgG antibodies. A two-tailed *p*-value < 0.05 was considered statistically significant.

## Results

### The overall occurrence and related major risk factors of *T. gondii* infection in studied ruminant

In the current study, *T. gondii* had an overall sero-occurrence of 29.5% (114/387) (χ^2^ = 1.532; *p* = 0.674). *T. gondii* antibodies were identified in 33.5% (52/155) of cattle, 28.2% (22/78) of buffaloes, 23.6% (21/89) of sheep, and 29.2% (19/65) of goats. In addition, no statistically significant variance (*p* > 0.05) was observed between the different species (Table 3).

Table 4 illustrates the seropositivity occurrence of *T. gondii* in relation to age, gender, breed, BCS, and location. The occurrence of anti-*T. gondii* antibodies were noticeably higher in female ruminants (46.1%) compared to males (17.1%) (χ^2^ = 20.371; *p* = 0.000). Additionally, animals > 5 years (38.6%) and between 2 and 5 years (34.8%) exhibited a significantly greater level of seropositivity compared to young animals (13.4%) (χ^2^ = 11.792; *p* = 0.0027). Regarding animal breeds, local breed animals showed a higher sero-occurrence for *T. gondii* (35.6%) than mixed breed animals (22.5%) (χ^2^ = 4.364; *p* = 0.0367). Also, the infection rate with *T. gondii* in poor body condition animals was significantly higher (52.3%) than in medium body condition animals (27.8%) and healthy animals (18.7%) (χ^2^ = 15.827; *p* = 0.0003). Significantly increased seropositivity was reported in animals from Edfu abattoir (39.7%), Kom Ombo (38.7%), and Daraw (21.8%) compared to those from Aswan abattoir (19.6%) (χ^2^ = 9.199; *p* = 0.0267). The variables under investigation in this study were found to be significantly associated with the incidence of *T. gondii* infection (*p* < 0.05), except for the comparison between species (*p* > 0.05).

**Table 3:** Sero-occurrence of *T. gondii* infection in studied ruminant

Species	Examined no.	Positives no.	Sero-occurrence, %	*p*-value	χ^2^
Cattle	155	52	33.5	0.674	1.532
Buffalo	78	22	28.2		
Sheep	89	21	23.6		
Goat	65	19	29.2		
Total	387	114	29.5		

**Table 4:** Risk factors related to *T. gondii* infection in studied ruminant

Variable	Category	Examined no.	Positives no.	Sero-occurrence (%)	*p*-value	χ2
Age	˂ 2 years	112	15	13.4	0.0027	11.792
	2–5 years	187	65	34.8		
	> 5 years	88	34	38.6		
Gender	Male	222	38	17.1	0.000	20.371
	Female	165	76	46.1		
breed	Local	205	73	35.6	0.0367	4.364
	Mixed	182	41	22.5		
BCS	Good	166	31	18.7	0.0003	15.827
	Medium	133	37	27.8		
	Poor	88	46	52.3		
Location	Aswan	143	28	19.6	0.0267	9.199
	Daraw	55	12	21.8		
	Kom Ombo	111	43	38.7		
	Edfu	78	31	39.7		

*BCS* Body condition score

*p *< 0.05 was regarded as the significant level

### Histopathological and IHC findings

Histopathological examination of tissue samples from 114 seropositive animals revealed that *T. gondii* tissue cysts were detected exclusively in 38 (33.3%) of the cardiac muscle specimens. In contrast, no cysts were identified in the diaphragm samples. A summary of species-specific histopathological and IHC findings is presented in Table 5.

Hematoxylin and eosin (H&E) staining demonstrated the presence of well-defined, round-to-oval-shaped tissue cysts within the cardiac muscle fibers. These cysts exhibited a distinct, smooth, and demarcated cyst wall encasing numerous small, crescent-shaped bradyzoites. The internal architecture of the cysts lacked any compartmentalization, consistent with the typical morphological features of *T. gondii* cysts.

**Fig. 2:**
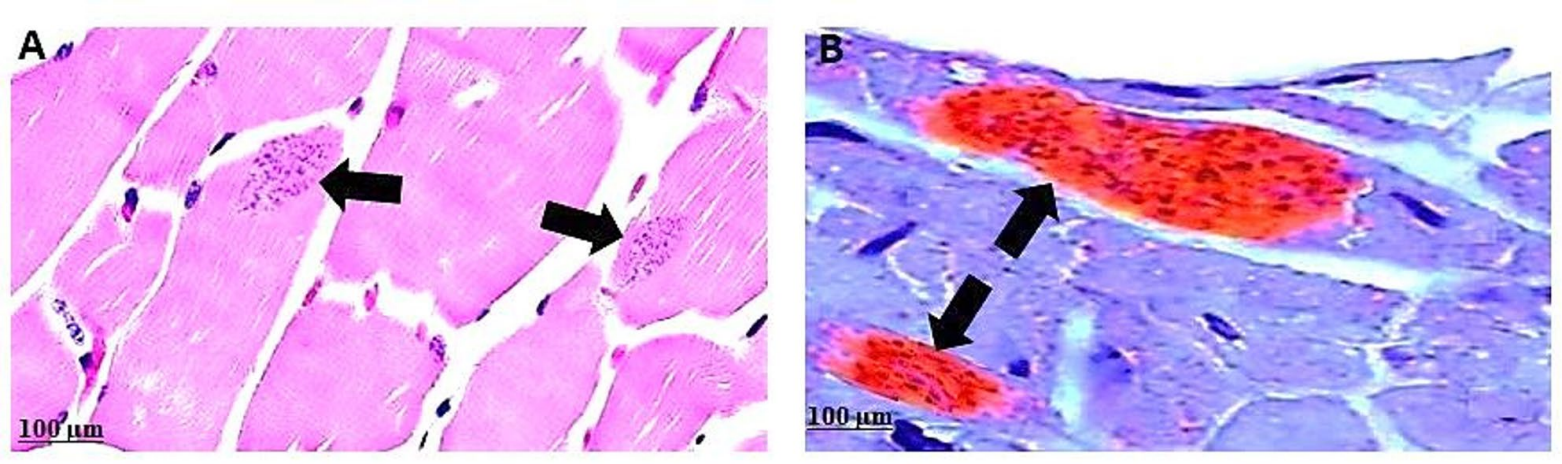
Histological sections of cardiac muscle showing *T. gondii* tissue cysts. **A** H&E staining reveals a well-circumscribed, round to oval-shaped tissue cyst embedded within cardiac muscle fibers. The cyst is filled with numerous small, crescent-shaped bradyzoites exhibiting characteristic basophilic (purple) staining. **B** IHC staining of the same tissue demonstrates strong positive immunoreactivity for *T. gondii* antigens. The presence of dense brown chromogenic granules within the cyst indicates a positive reaction, confirming the identity of *T. gondii* tissue cysts

**Fig. 3:**
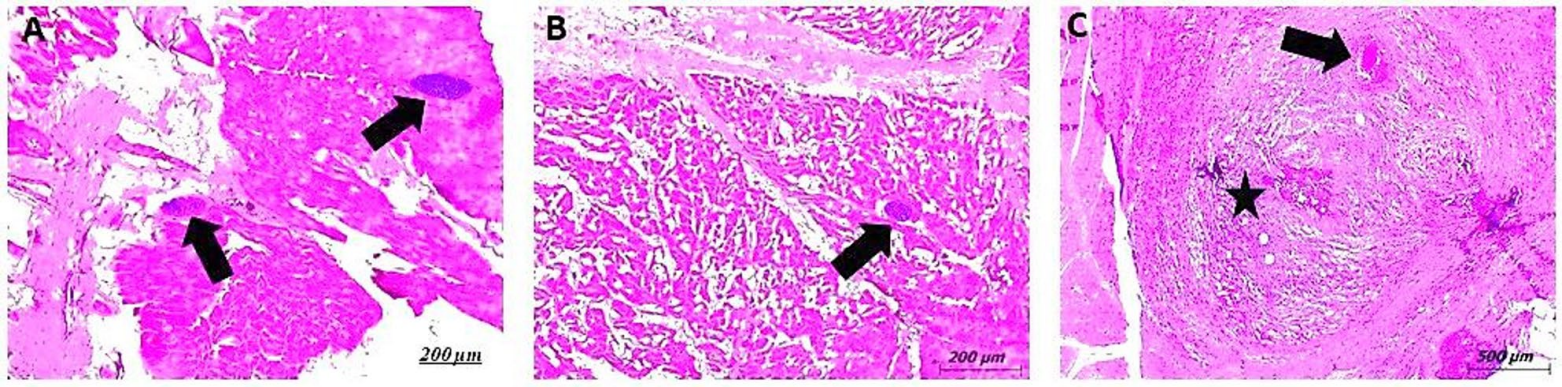
Photomicrographs of cattle skeletal muscle tissue sections stained with H&E, illustrating pathological alterations associated with *T. gondii* infection. **A**, **B** Sections show focal areas of muscle fiber necrosis and degeneration, accompanied by mixed inflammatory cell infiltration. A well-demarcated *T. gondii* tissue cyst (arrow) is observed, embedded within the affected muscle tissue. **C** Displays varying degrees of inflammatory cell infiltration (star), including granulomatous inflammation (arrow). The surrounding muscle fibers exhibit marked degenerative changes, including loss of cross-striations and fiber disintegration, resulting in the disruption of normal muscle architecture

Surrounding the cysts, areas of muscle tissue necrosis were evident, often accompanied by varying degrees of inflammatory cell infiltration. The infiltrates were composed predominantly of eosinophils and macrophages, indicating a chronic inflammatory response. Additionally, increased deposition of collagen fibers was observed, indicating ongoing tissue remodeling and fibrosis. The adjacent cardiac muscle fibers exhibited marked degenerative changes, including loss of cross-striations, vacuolar degeneration, and fragmentation, ultimately leading to disruption of the normal muscle architecture. These findings highlight the localized nature of *T. gondii* tissue cyst formation in cardiac muscle, as well as the associated pathological alterations, including inflammation, necrosis, and myofiber degeneration (Figs. 2A and 3).

IHC analysis was performed to confirm the occurrence of *T. gondii* cysts in the cardiac muscle tissues of seropositive specimens. The examined sections demonstrated a strong positive immunoreactivity, characterized by intense brown chromogenic staining localized within the cytoplasm of lymphoblast-like inflammatory cells within the cysts. The brown deposits, indicative of the DAB (3,3’-diaminobenzidine) substrate reaction, provided clear evidence of the presence *T. gondii* antigen within the infected tissues. Notably, the immunolabeling was highly specific, with no significant background staining observed in the surrounding non-infected tissue areas. This positive IHC reaction confirmed the histologically identified cysts as *T. gondii* and supported the diagnosis of toxoplasmosis (Fig. 2B).

**Table 5:** A summary of species-specific histopathological, IHC, and PCR findings

Species	No. seropositive animals examined	No. with cysts detected in cardiac muscle (%)	No. with cysts detected in diaphragm (%)	IHC confirmation (%)	No. PCR-positive (%)
Cattle	52	22 (42.3%)	**0**	22 (42.3%)	52 (100%)
Buffalo	22	8 (36.4%)	**0**	8 (36.4%)	22 (100%)
Sheep	21	4 (19%)	**0**	4 (19%)	21 (100%)
Goat	19	4 (21.1%)	**0**	4 (21.1%)	19 (100%)
Total	114	38 (33.3%)	**0**	38 (33.3%)	114 (100%)

#### Molecular findings

**Fig. 4:**
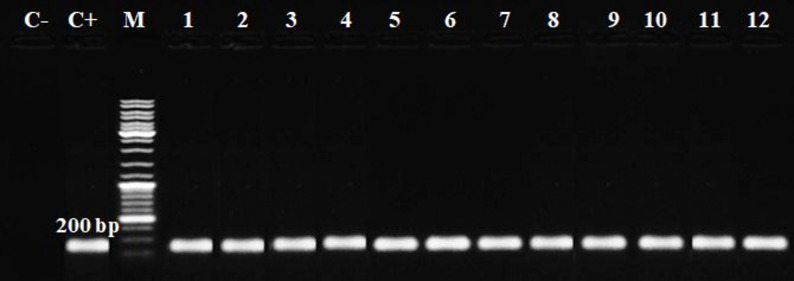
The first round of PCR amplification uses the outer pair of primers targeted to the *B1* gene. The amplicon size of 200 bp in length denotes *T. gondii*. M: DNA ladder; C+: positive control; C-: negative control. Lanes 1–3: cattle, Lanes 4–6: buffaloes, Lanes 7–9: sheep, and Lanes 10–12: goat

**Fig. 5:**
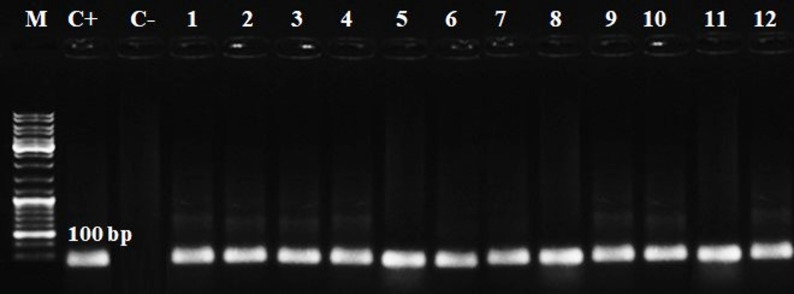
Agarose gel electrophoresis for analysis of nested PCR for *T. gondii* identification at 100 bp. M: DNA ladder; C+: positive control; C-: negative control. Lanes 1–3: cattle, Lanes 4–6: buffaloes, Lanes 7–9: sheep, and Lanes 10–12: goat

Molecular analysis confirmed the occurrence of the *T. gondii* B1 gene in all seropositive blood samples (*n* = 114) by nested PCR at 100 bp (Figs. 4 and 5). When analyzed by host species, PCR positivity was 100% across cattle (*n* = 52), buffalo (*n* = 22), sheep (*n* = 21), and goats (*n* = 19). A summary of PCR positivity is presented in Table 5.

## Discussion

Toxoplasmosis presents substantial clinical and economic challenges to both human and animal health. It usually presents as signs of the flu in people, such as fever, lymphadenopathy, and eye problems. The infection can also cause a variety of neurological and reproductive diseases [2]. In livestock, it is a significant contributor to abortion, stillbirth, and poor offspring, all of which have a detrimental effect on economic production and sustainability [37]. Due to the significant health impacts on both humans and animals, regularly updating data on *T. gondii* in various hosts is crucial for establishing a standard for its management. The study offers current information regarding the distribution of *T. gondii* in domestic ruminants across the nation (Table 1). Nonetheless, comparisons between researches should be made with precaution due to variations in the number of animals studied, serological and molecular techniques used, and handling and environmental conditions. Toxoplasmosis subclinically exists in domestic ruminants, complicating disease identification based solely on clinical performance. Therefore, laboratory-based techniques that provide information on the incidence of *T. gondii* infection in these animals are the only way to determine the risk of spreading to humans from raw and undercooked meat [38].

In the current study, we determined the seroprevalence of *T. gondii* in several locations of the Aswan governorate, revealing a varied geographical spread and susceptibility to this parasite throughout the study area. Taking this into consideration, serological surveys are significant diagnostic techniques used to monitor diseases like *T. gondii*. They are thought to offer definitive evidence for sufficient toxoplasmosis monitoring [39]. The high individual sero-occurrence value of *T. gondii* in cattle (33.5%), goats (29.2%), buffalo (28.2%), and sheep (23.6%) in our study did not show a significant relationship between the examined species (χ^2^ = 1.532; *p* = 0.674). This suggests that the parasite is prevalent in Upper Egypt, which could be a concern for public health and animal welfare. Results for the total seroprevalence of 29.5% are consistent with those from other reports of this protozoon in Egypt (Table 1). Several factors could contribute to the higher seroprevalence rate in small ruminants compared to large ruminants. One possible explanation is that small ruminants typically feed themselves by grazing on pastures, while large ruminants usually remain indoors. Another factor is the occurrence of cat, which can contaminate feed and water, increasing the risk of infection [40]. In this study, we detected *T. gondii* less frequently in buffalo (28.2%) than in cattle (33.5%). The lower detection rate in buffalo compared to cattle may be related to the animals’ feeding behaviors and variations in susceptibility to *T. gondii* [1].

Nevertheless, the seroprevalence rate of *T. gondii* in our investigation matches the rates earlier identified in both small and big ruminants in numerous Egyptian governorates (Table 1), although levels varied widely among studies. However, the *T. gondii* in ruminants has been studied in multiple countries around the world, such as Malaysia, where it was found in 25% of the samples, with 9.1% in cattle, 54.7% in goats, and 34.9% in sheep [7]; in Iran, where it was 11.6% overall, with 14.4% in sheep and 8.8% in goats [9]; in Poland, where the rate was 13.0% in cattle [10]; in Italy, where it was 63.3% in goats [11]; and in Algeria, where it was found in 53.26% of goats [40].

In a prior investigation in Lower Egypt, investigators detected higher rates of infection in cattle (59.4%), buffalo (58.6%), sheep (38.8%), and goats (46%), using a similar diagnostic technique to the current research. The aforementioned findings in Table 1 revealed significant differences according to the serological test methodology. MAT is one of the most widely accepted approaches for detecting *T. gondii* infection in both animals and people [41]. The test offers several advantages over other serological techniques and is widely used due to its validity and effectiveness in detecting antibodies against the parasite. Additionally, MAT is recognized for being easy to use, affordable, fairly accurate, and highly sensitive, which makes it a popular choice for detecting parasites in various animal species [2]. However, it should be noted that indirect ELISA techniques, which use pure tachyzoite lysates or recombinant antigens, have been shown to have greater sensitivity and specificity in a variety of host species (e.g., cattle, sheep, goats). ELISA also offers advantages for large-scale epidemiological surveys due to its low subjectivity in result interpretation and its potential for automation [42]. Nevertheless, MAT was chosen for the current study due to its ability to be used across multiple animal hosts and its lack of species-specific conjugates, which renders it particularly well-suited for multi-species investigations like ours [2]. Subsequent research in Egypt may enhance diagnostic precision and facilitate more comprehensive sero-epidemiological assessments by integrating MAT with ELISA.

The lower prevalence in the current study compared to other governorates, especially those in Lower Egypt, such as Alexandria, Cairo, Al-Sharkia, and Dakahlia, may be influenced by numerous factors related to climatic differences, including temperature and humidity. In comparison to other Aswan environments, Lower Egypt is generally characterized by excessive moisture and relatively lower temperatures [43]. These environmental factors may help the oocysts survive longer in the surroundings, which raises the chances of coming into contact with the parasite. Additionally, the likelihood of *T. gondii* transmission to other associated species is likely increased by the larger number of definitive hosts, notably cats, in the densely populated areas of Lower Egypt, particularly Cairo. Additionally, ruminant management in some governorates relies on extensive grazing practices with minimal biosecurity measures [2]. Variations in the number of main animal hosts, the size of samples taken, the blood testing methods used, and the limits and accuracy of tests also affect these differences [44].

It is important to note that the statistical analysis confirmed that age, gender, breed, BCS, and location were independent predictors of seropositivity, supporting their epidemiological importance (Table 3). In this regard, ruminants over the age of five had a greater *T. gondii* (38.6%) than did young animals (13.4%). This result is consistent with prior studies showing that older animals are more exposed to *T. gondii*, which causes their antibody levels to rise with age [25]. Additionally, adult animals have been around longer and encounter more sources of infection than younger ones, which explains why older animals have higher rates of *T. gondii* [2]. The data show that the occurrence rate is larger in females (46.1%) than in males (17.1%). This difference is explained by the fact that males are more likely to undergo fattening and meat production and be delivered for slaughter more quickly. At the same time, females are retained in the herd for longer periods to reproduce and lactate, which increases their chances of contracting the infection [45]. The current study found that native breeds have a higher infection rate than imported breeds, likely due to uncontrolled movements of livestock and shared grazing areas for local breeds, as well as differences in their susceptibility to *T. gondii* infection [45].

The research findings validated the concept that an animal’s body state is positively correlated with the chance of *T. gondii* infection. Additionally, they showed that the occurrence was higher in animals with poor body conditions than in those with medium and good body conditions, suggesting that *T. gondii* infection is a persistent and incapacitating condition that results in the progressive loss of body mass, especially in infected animals with other parasitic infections [40]. The risk factor analysis showed that animals from Edfu and Kom Ombo were more likely to be exposed to *T. gondii* than those in the Daraw and Aswan areas, indicating that this parasite is more common in those regions. The animals in the areas under investigation were reared in an indoor smallholder farming system, with or without adequate biosecurity, and companion and stray cats had unrestricted access to the cattle’s housing, feed, and drinking water. This behavior of cats roaming freely increases the likelihood of *T. gondii* infection by consuming sporulated oocysts or tissue cysts from other animals. Thus, stray cats could be one of the factors impacting *T. gondii* circulation in this region [46].

Methods for diagnosing livestock must be improved [47]. In this respect, this research exposed the heart and diaphragm tissues from seropositive slaughtered carcasses to a histopathological study to examine the presence of *T. gondii* cysts. It revealed the presence of only tissue cysts in the heart tissues. In the current investigation, the incidence of histopathological variations was considered low compared to serology and molecular approaches, as previously reported in studies that highlighted the challenges of identifying parasites through the histological approach [48, 49]. This condition can be attributed to several factors, including the low number of cysts in muscular tissues and the non-uniform distribution of parasites. It is also crucial to note that the sensitivity rate of the histopathological technique is lower in naturally infected animals, as was the case with some animals in the current study [49].

The histological examination of the animal tissues under research showed *Toxoplasma* tissue cysts that are highly well-defined, with a typical round-oval cyst wall filled with many tiny bradyzoites and no partitioning within the cyst. Furthermore, there are sections of tissue necrosis that demonstrate various levels of inflammatory infiltrate and collagen fibers, with a particular emphasis on eosinophils and macrophages. Additionally, the muscle fibers go through degenerative changes that may result in the loss of their normal structure (Figs. 2 and 3). Protozoa are present in cysts within muscle fibers, providing defense against host immunity—a theory that has been proven for a variety of parasites—and so supporting the apparent lack of inflammatory reaction in certain tissues [50].

Previous studies classified lesions into several stages based on the degree of immune response. The process begins with a living parasite encased in a delicate layer of collagen. Subsequently, mononucleotide inflammatory cells launch an assault on the parasite. Eventually, granulomatous tissue is produced, its centers housing amorphous substances. The destroyed parasite is then in a state of chronic infection [51]. This is in line with current research, which shows that immune cells infiltrate lesions with a layer of collagen fibers, while other lesions have dense layers of connective tissue and a high number of fibroblasts. In addition, some reactor animals may have lacked pathology due to the short time between infection and postmortem examinations [52], which explains the lower histopathological percentage compared to the serology findings. However, some researchers found that histopathology was an effective approach for detecting coccidian tissue cysts in bovine muscles [5].

The IHC procedure is recognized for its precision in labeling and localizing the distribution and quantity of a specific molecule in cells and tissues through the use of particular reactions between antigens and antibodies. It is done in a way that preserves the tissue structure, allowing us to observe how the molecule is expressed in its natural environment [47, 53]. In addition, the heart might be the most effective organ for using IHC to identify *T. gondii* infection [54, 55]. In the current research, we found positive signs of *T. gondii* in the heart tissue from slaughtered animals that tested positive, which agrees with findings from other researchers [53, 54]. However, another study was unable to identify the cysts or tachyzoites of *T. gondii* in the tissues they examined through IHC [56]. The differences in these outcomes may be due to the varying levels of infection in the animals and their unique physical and immune conditions; additionally, the accidental manner in which the parasites are spread could also play a role [56]. IHC is a helpful technique for detecting *T. gondii* in animal tissues, as it identifies the parasites both in animals with no obvious infection by traditional histopathology and in those with low serum titers of *T. gondii*-specific antibodies [35].

The polymerase chain reaction (PCR) is established on the in vitro amplification of parasite-specific DNA sequences, which are visualized by agarose gel electrophoresis. In addition, nested PCR is a technique for the multiplication (replication) of DNA samples that uses two sets of PCR primers to amplify fragments. The first pair of primers (external) amplifies the fragment, which works similarly to single PCR in general. The second pair of primers (internal) is referred to as nested primers because they attach to the first PCR product and help generate a shorter second PCR product [14].

In the present work, a single amplicon with a predicted size was amplified by first-round single PCR (external and internal primers) and nPCR *B1* gene primers (Figs. 4 and 5). nPCR (*B1*) achieved in this research indicates the occurrence of *T. gondii* DNA in the blood samples of seropositive ruminants. Numerous investigations have demonstrated the excellent sensitivity of nPCR targeting the *B1* gene to identify *T. gondii* DNA in ruminants [7]. The PCR products in this study were found to be identical to those reported in earlier studies in Iran [1], Poland [10], Indonesia [14], and Italy [57]. In contrast, in Malaysia failed to detect any *T. gondii* DNA in any of the goat and sheep samples [7]. The *B1* gene was chosen as the marker for several reasons: it has the largest collection of available sequences covering a diverse range of isolates; many studies utilize this gene for identifying toxoplasmosis; it is precise and accurate for detecting *T. gondii*, as ribosomal DNA is often repeated within eukaryotes; there are approximately 35 copies in the *T. gondii* genome, and it has historically been a reliable PCR target [14].

Serological techniques are useful for determining the frequency of exposure to specific pathogens. Still, they are unable to differentiate between an infection that has already occurred and one that is now happening, nor can they demonstrate tissue infectivity. On the other hand, the identification of tissue cysts by IHC in cardiac muscle specimens and the PCR detection of *T. gondii* DNA in all seropositive animals provide clear proof of infection. These findings have significant implications for food safety, as tissue cysts represent infectious phases that may persist in meat products consumed by people [55, 57]. Previous research has also emphasized the need for molecular and histological confirmation to determine the risk of transmission through meat consumption [5, 14, 53]. Our findings provide a more comprehensive and reliable assessment of the threat posed by *T. gondii* to ruminant populations in Upper Egypt, achieved by combining IHC and PCR with serology.

It is essential to acknowledge that this study has several limitations. First, the sampling technique was abattoir-based and not intended to be entirely representative of the area’s ruminant populations; consequently, our data should be viewed as representing the occurrence rather than the actual prevalence of *T. gondii* infection. Second, although several host and management variables were statistically associated with seropositivity, the analysis was limited to univariate chi-square testing, and the absence of multivariate modeling may reduce the ability to disentangle confounding effects. Third, the molecular detection was dependent on nested PCR that targeted the *B1* gene. Even though *B1* has been studied extensively, research has demonstrated that the *REP529* repeat element is more sensitive in identifying low parasite loads [38].

Furthermore, nested PCR has an inherent risk of amplicon contamination, which might jeopardize specificity if proper safeguards are not taken. In addition, we note that IgG seropositivity indicates earlier exposure to *T. gondii* rather than necessarily active infection. Thus, statistical associations reported here refer to exposure (serostatus). Molecular detection and histopathology were used to confirm infection in seropositive animals; however, formal risk-factor analyses were not performed using molecular or histopathological results, as these tests were applied only to seropositive samples.

## Conclusion

This work presents a recent comprehensive analysis of *T. gondii* infection and related risk factors in ruminant populations in Aswan, Upper Egypt, based on evidence from serology, molecular detection, and histopathology. Our research revealed the presence of *T. gondii* among slaughtered ruminants in the Aswan governorate. Detecting parasite DNA in blood samples and tissue cysts in cardiac muscle confirms that animals entering the food chain are infected, highlighting a potential risk of zoonotic transmission through the consumption of raw or undercooked meat. Strengthening meat safety practices, public awareness, and surveillance programs is recommended to mitigate the public health impacts of toxoplasmosis. Ruminant rising, alongside the implementation of suitable immunization programs, should be considered to minimize the risk of ruminant exposure to this zoonotic parasite. More extensive blood tests and genetic studies are necessary to understand how these animals contribute to the spread of *T. gondii* in Egypt and to investigate the possible connection to human cases, as well as the genetic similarities of the strains found.

## Supplementary Information

Supplementary Material 1.

## Data Availability

The manuscript included all our available data.
