# Supplementary figures and images for: Seromolecular and histopathological study on Toxoplasma gondii infection in ruminants in Aswan, Egypt
Source: BMC Vet Res. 2025 Dec 30;22:49. doi: 10.1186/s12917-025-05195-9 (PMC12849353; doi:10.1186/s12917-025-05195-9)

Raw PCR Images

**
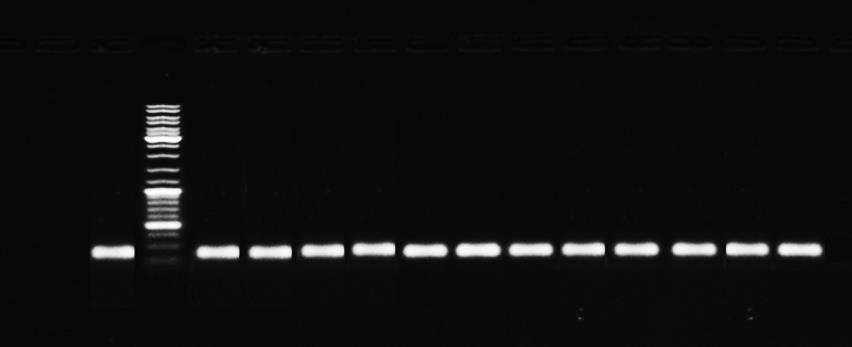
**

**
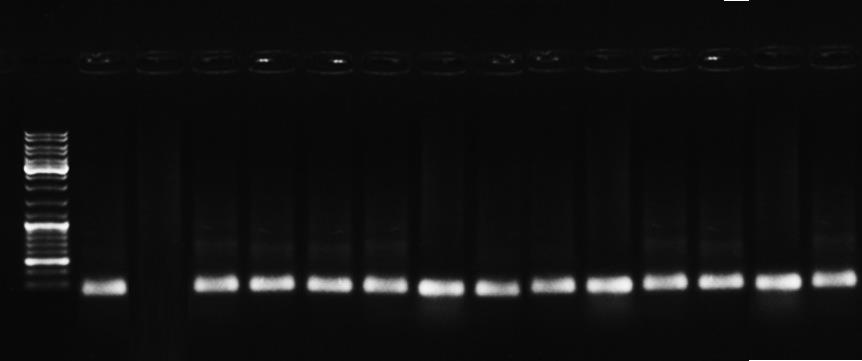
**

Supplement: Supplementary file 1 — Supplementary Material 1. [file 12917_2025_5195_MOESM1_ESM.docx]
